# The 2026 pulmonary embolism guideline in a regional intensive care unit: Reading the recommendations without a catheter laboratory

**DOI:** 10.1016/j.ccrj.2026.100218

**Published:** 2026-09-09

**Authors:** Prashant Maan

**Affiliations:** a Intensive Care Unit, Hervey Bay Hospital, Wide Bay Hospital and Health Service, Queensland, Australia; b University of Queensland, Queensland, Australia

**Keywords:** Pulmonary embolism, Thrombolysis, Regional intensive care, Reperfusion, Health services accessibility

## Abstract

The 2026 American Heart Association/American College of Cardiology pulmonary embolism guideline sets out a severity-based framework and a range of reperfusion strategies, including catheter-directed and mechanical therapies that most regional intensive care units in Australia cannot deliver on site. For the lowest-risk and the most severe high-risk patients, management is largely determined by severity and is similar wherever the patient presents. The difficult group is the patient with intermediate-high or early high-risk pulmonary embolism who may deteriorate but does not yet warrant immediate thrombolysis, and who often carries a bleeding risk that thrombolysis would worsen. The absence of on-site advanced therapy matters most for this group. The guideline helps here by setting out the available therapies and their limits and by defining when to involve a tertiary centre. Systemic thrombolysis remains the only on-site reperfusion option for a regional unit, and for the deteriorating patient without prohibitive bleeding risk, it is a reasonable choice consistent with current best-available evidence. We set out how a regional unit might reason through these decisions and offer a management table as one worked example. The table is a local adaptation and has not been prospectively validated.

The 2026 American Heart Association/American College of Cardiology multisociety guideline for acute pulmonary embolism sets out a severity-based classification and a range of reperfusion strategies.^1^ Several of these, including catheter-directed thrombolysis and mechanical thrombectomy, require an interventional suite and subspecialty services that most regional intensive care units in Australia do not have. The nearest tertiary centre may be several hundred kilometres away, with retrieval times of 4–6 h or more from the decision to transfer to the patient's arrival. Systemic thrombolysis is the only advanced reperfusion therapy that can be delivered locally, and it can be delivered within minutes.

The guideline itself addresses the situation of a centre without on-site advanced therapy. It advises referral to a tertiary centre where appropriate, but also states that patients who are unsuitable for transfer should be treated according to the best available local expertise, and that thrombolysis should not be delayed before transfer.^1^ The real difficulty for a regional unit is not the shocked patient, who needs prompt thrombolysis wherever they present, nor the low-risk patient, who can be anticoagulated safely. It is the patient who may deteriorate but does not yet meet criteria for emergent thrombolysis, and who often carries a bleeding risk that thrombolysis would worsen. This is the group this article is mainly concerned with, and the group in whom distance, cost, and procedural exposure bear most on the decision.

For that group, the principle we set out is a cautious one. In a patient without prohibitive bleeding risk, there is no evidence that catheter-based or device therapy produces better patient-centred outcomes than systemic thrombolysis, because the two have never been compared directly. A regional unit can therefore anticoagulate and monitor closely, give systemic thrombolysis locally when the trajectory indicates deterioration, and reserve transfer for the patient in whom systemic thrombolysis is contraindicated or hazardous and whose trajectory is worsening. A regional centre practising this way is acting in line with current best-available evidence. The sections that follow set out the evidence for each part of this approach and the points where that evidence runs out.

No reperfusion strategy in pulmonary embolism carries a class 1 recommendation in any risk category.^1^ Systemic thrombolysis in the highest-risk patient sits at class 2a (reasonable to administer) with a level of evidence of C-LD (limited data). The mortality evidence for thrombolysis in high-risk pulmonary embolism rests on a single randomised trial of eight patients per arm, stopped early, using streptokinase rather than a contemporary agent,^2^ together with registry data and physiological reasoning. Every reperfusion recommendation above anticoagulation alone is built on limited or nonrandomised data. This is the evidentiary floor on which all subsequent comparisons stand, and it is low.

Catheter-directed thrombolysis and mechanical thrombectomy have never been tested against systemic thrombolysis in a randomised trial in any risk category. The device trials that inform current practice compared their device against anticoagulation alone, or against another catheter strategy, but not against the drug given systemically. Higher-risk Pulmonary Embolism Thrombolysis (HI-PEITHO) compared ultrasound-facilitated catheter-directed alteplase against anticoagulation alone.^3^ STORM-PE compared mechanical thrombectomy against anticoagulation alone.^4^ PEERLESS compared large-bore thrombectomy against catheter-directed thrombolysis, with neither systemic thrombolysis nor anticoagulation alone as a comparator.^5^ None of these trials can tell a clinician whether a device is better, worse or equivalent to systemic thrombolysis, because that comparison was not made. What the evidence from Pulmonary Embolism Thrombolysis (PEITHO), HI-PEITHO, STORM-PE and STRATIFY does suggest is that restoring pulmonary blood flow earlier, by whatever means, may reduce cardiorespiratory decompensation compared with anticoagulation alone in selected higher-risk patients who do not yet meet criteria for emergent thrombolysis. For these patients, the question is which reperfusion to use: systemic thrombolysis on site, or a device-based approach that, in a regional centre, requires transfer.

The STRATIFY trial is informative here, because it isolated the variable that device trials leave confounded. It gave the same 20 mg dose of alteplase by two routes, ultrasound-assisted catheter delivery and peripheral intravenous infusion, and compared both against heparin.^6^ Alteplase by either route reduced clot burden more than heparin, and the catheter and ultrasound added nothing measurable over the same drug given through a peripheral line. Because pulmonary blood flow is high, a thrombolytic given into the pulmonary artery disperses systemically almost at once, so the rationale for local delivery was always weak. STRATIFY used a surrogate endpoint, clot burden on computed tomography, and was not powered for clinical outcomes, so it shows that the route of delivery does not change the drug's effect without proving clinical benefit. If the catheter contributes nothing over peripheral delivery of the same drug, the positive results of the device trials are attributable to the drug and not to the device.

The bleeding risk of thrombolysis is not small, and it falls hardest on exactly the patients a regional unit finds most difficult. In PEITHO, full-dose tenecteplase in intermediate-risk patients increased major extracranial bleeding compared with anticoagulation alone, 6.3% versus 1.2%, and haemorrhagic stroke, 2.0% versus 0.2%.^7^ These are the patients who would fall into the C3 and D1 categories of the new classification, and in intensive care, they are often the patients whose bleeding risk is already raised by trauma, recent surgery, or complex medical illness. The major device and thrombolysis trials excluded patients with a contraindication to anticoagulation, a contraindication to thrombolysis, or a high bleeding risk, so the group in whom the decision is hardest is the group the trials left out. The trial evidence does not cover these patients, and any framework applied to them is an extrapolation.

There is an intuition that devices should cause less bleeding than systemic thrombolysis. This is reasonable, but it has not been tested against systemic thrombolysis, and it leaves out the procedural hazards that devices introduce: femoral puncture with the risk of inguinal and retroperitoneal haemorrhage, sedation, a period of lying flat, and transport to a catheter suite away from intensive care monitoring at the point in the illness when a patient may deteriorate. Systemic thrombolysis avoids these, but it carries its own consequence that is easy to overlook: once a patient has been thrombolysed, any subsequent invasive procedure carries a higher bleeding risk, including the central venous and arterial catheters that a critically ill patient routinely needs. The adverse-event profile of the newer devices is not yet well described. Both paths carry risk that have not been quantified against the other, and a regional unit without these devices has not been shown to be at a safety disadvantage.

The pulmonary embolism response team carries a class 1 recommendation.^1^ Its documented effects are largely on process and morbidity rather than survival: faster time to therapeutic anticoagulation, reduced use of inferior vena cava filters, more consistent management, and shorter hospital and intensive care lengths of stay. Several of these are patient-centred outcomes in their own right, and length of stay carries its own morbidity. A mortality benefit has not been demonstrated, so a centre without a response team is not lacking an intervention shown to save lives. The main value of a response team is structured multidisciplinary decision-making for the higher-risk, higher-bleeding patients discussed here. A regional unit cannot build a full response team. A regionalised service, in which a regional or smaller metropolitan unit shares the decision with a tertiary centre, could bring that structured decision-making to the patients who most need it and is worth considering as these services develop.

The reperfusion evidence does not clearly favour the resourced centre over the regional one. The 2026 American Heart Association/American College of Cardiology guideline does not raise the quality of that evidence, which remains limited. It does provide a framework, and in two respects that framework helps a regional unit. First, it defines an intermediate stage between the stable intermediate-high-risk patient and frank shock. Transient hypotension and normotensive shock, marked by a normal or near-normal blood pressure together with a sign of hypoperfusion such as a raised lactate, oliguria, acute kidney injury, altered mental status or a low cardiac index, are recognised as a distinct stage at which thrombolysis may be considered.^1^ This gives a defined point at which to treat on trajectory, before established shock, which is the window in which a regional unit can act in without transfer. It is consistent with PEITHO, in which most placebo-arm patients who deteriorated were rescued with thrombolysis at that later point and most survived.^7^ Second, the guideline states that transferring an unstable high-risk patient before stabilisation is harmful,^1^ which supports treating locally when transfer is unsafe. For a regional unit, the guideline sets out which therapies exist and where their limits lie, and it gives defined points at which to treat locally and at which to seek tertiary referral.

The absence of a head-to-head comparison is not the same as evidence that catheter-based therapies do not work. These are relatively new therapies, and their evidence base is still developing. The trajectory may resemble that of coronary angiography and endovascular clot retrieval, which moved from uncertain to standard as trial evidence accumulated. The PE-TRACT trial, an investigator-initiated study funded by the United States National Institutes of Health, is comparing catheter-directed therapy with anticoagulation alone in intermediate-risk patients, with long-term functional outcomes as its focus,^8^ and trials of this kind may eventually clarify which patients benefit from device-based reperfusion. Until they report, there is no comparative evidence to guide the choice between systemic thrombolysis and transfer for a device, and the choice is a matter of clinical judgement.

That judgement turns on bleeding risk, and the bleeding-risk side deserves more scrutiny than it usually receives. The contraindications to thrombolysis in pulmonary embolism mirror those used in acute ischaemic stroke and myocardial infarction, and have not been separately validated in a pulmonary embolism population. They are presented as absolute or relative, but that division is not itself grounded in pulmonary embolism outcome data. As the risk of death from the embolism rises, the threshold for accepting bleeding risk shifts, so the boundary between an absolute and a relative contraindication behaves as a sliding risk-benefit judgement and not a fixed rule. The patient who stands to benefit most from thrombolysis is the one with the highest risk of death from pulmonary embolism and the lowest risk of bleeding; the patient who stands to benefit least, and may be harmed, is the reverse. The stratification that guides this is not backed by hard evidence for where the lines should sit, and the C3 and D1 patients, at risk of both deterioration and bleeding, are not well served by a single automatic rule.

This defines the patient for whom transfer, or at least a shared decision with a tertiary centre, is most worth considering: the C3 to D1 or D2 patient with a bleeding risk that makes systemic thrombolysis hazardous, who is deteriorating along a trajectory that will require reperfusion, and who is stable enough to survive retrieval. For this patient, a catheter-based approach may be worth its procedural risk, because the safer alternative is not readily available on site. The regional unit should involve the tertiary centre early, while the patient can still be moved, rather than waiting until deterioration makes transfer unsafe. Outside this group, transfer adds hours of retrieval time and procedural risk without demonstrated benefit, and the patient is generally better served by treatment on site.

The approach that follows from this reasoning is set out in Table 1, organised by risk category and by bleeding risk. It is offered as a reasoned local adaptation for a centre whose only on-site reperfusion option is systemic thrombolysis, not as a validated protocol. The C3 and D1 rows, in particular, are prompts to multidisciplinary discussion, not fixed instructions. No component of it has been prospectively tested, for the same reason that the guideline recommendations it draws on rest on limited evidence: the trials that would validate it have not been done. Clinicians in these units are already making these decisions, with or without a framework to structure them, and a reasoned local approach is preferable to an ad hoc one.

**Table 1:** A regional intensive care approach to pulmonary embolism by risk category and bleeding risk, for a centre whose only on-site reperfusion option is systemic thrombolysis. The C3 and D1 rows are prompts to multidisciplinary discussion, not fixed instructions.

Category	No bleeding risk	Relative bleeding risk	Absolute contraindication
C3	Anticoagulate. Monitor 24–72 h. Multidisciplinary discussion. Rescue thrombolysis if deterioration develops	Anticoagulate. Multidisciplinary discussion. Rescue half-dose thrombolysis if deterioration develops	Anticoagulate. Multidisciplinary discussion. Early call to tertiary centre if trajectory worsening
D1/D2	Full-dose thrombolysis (multidisciplinary discussion at D1)	Half-dose thrombolysis (multidisciplinary discussion at D1)	Transfer to tertiary centre urgently. Rescue thrombolysis if hypotensive en route
E1	Full-dose thrombolysis	Half-dose thrombolysis. Can repeat once if no response	Half-dose thrombolysis. Can repeat if needed
E2	Full-dose thrombolysis immediately	Full-dose thrombolysis	Full-dose thrombolysis

Categories follow the 2026 AHA/ACC Acute Pulmonary Embolism Clinical Categories. C3, elevated clinical severity score with raised troponin and right ventricular dysfunction; D1, transient hypotension; D2, normotensive shock; E1, persistent hypotension; E2, refractory hypotension or cardiac arrest. Half-dose thrombolysis refers to reduced-dose systemic thrombolysis. This table is a local adaptation and has not been prospectively validated.

The conclusion for regional practice is measured. For the patient without prohibitive bleeding risk, the regional approach is anticoagulation, close monitoring, and prompt systemic thrombolysis when deterioration declares itself. This is in line with current best-available evidence. For the harder group, at risk of both deterioration and bleeding, the decision is genuinely uncertain and belongs in a shared discussion with a tertiary centre. The 2026 guideline is useful to regional and smaller metropolitan centres here. It provides a framework for recognising these patients and for referring for advanced reperfusion therapy when thrombolysis would carry prohibitive risk.

## Author Contribution

Prashant Maan: Conceptualization, Writing – original draft, Writing – review & editing.

## Conflicts of interest

The authors declare that they have no known competing financial interests or personal relationships that could have appeared to influence the work reported in this paper.
